# Sap fluxes from different parts of the rootzone modulate xylem ABA concentration during partial rootzone drying and re-wetting

**DOI:** 10.1093/jxb/erv029

**Published:** 2015-03-04

**Authors:** J. G. Pérez-Pérez, I. C. Dodd

**Affiliations:** ^1^Department of Citriculture, IMIDA, 30150 La Alberca, Murcia, Spain; ^2^Centre for Sustainable Agriculture, Lancaster Environment Centre, University of Lancaster, Lancaster LA1 4YQ, Uk

**Keywords:** ABA, irrigation scheduling, partial rootzone drying, root-to-shoot signalling, soil moisture sensors, soil moisture heterogeneity.

## Abstract

Previous studies with partial rootzone drying (PRD) irrigation demonstrated that alternating the wet and dry parts of the rootzone (PRD-Alternated) increased leaf xylem ABA concentration ([X-ABA]_leaf_) compared with maintaining the same wet and dry parts of the rootzone (PRD-Fixed). To determine the relative contributions of different parts of the rootzone to this ABA signal, [X-ABA]_leaf_ of potted, split-root tomato (*Solanum lycopersicum*) plants was modelled by quantifying the proportional water uptake from different soil compartments, and [X-ABA]_leaf_ responses to the entire pot soil-water content (θ_pot_). Continuously measuring soil-moisture depletion by, or sap fluxes from, different parts of the root system revealed that water uptake rapidly declined (within hours) after withholding water from part of the rootzone, but was rapidly restored (within minutes) upon re-watering. Two hours after re-watering part of the rootzone, [X-ABA]_leaf_ was equally well predicted according to θ_pot_ alone and by accounting for the proportional water uptake from different parts of the rootzone. Six hours after re-watering part of the rootzone, water uptake by roots in drying soil was minimal and, instead, occurred mainly from the newly irrigated part of the rootzone, thus [X-ABA]_leaf_ was best predicted by accounting for the proportional water uptake from different parts of the rootzone. Contrary to previous results, alternating the wet and dry parts of the rootzone did not enhance [X-ABA]_leaf_ compared with PRD-Fixed irrigation. Further work is required to establish whether altered root-to-shoot ABA signalling contributes to the improved yields of crops grown with alternate, rather than fixed, PRD.

## Highlight

Following drying and re-wetting events during partial rootzone drying irrigation, xylem ABA concentration was best explained by accounting for sap fluxes from both parts of the rootzone.

## Introduction

Soil moisture varies considerably both temporally (due to rainfall events or irrigation of crop plants) and spatially (roots usually dry the surface soil layers while considerable moisture may be available at depth), and both plant fitness and crop productivity depend on the root system capturing sufficient water to sustain growth. There has been considerable agronomic interest in different irrigation techniques such as partial rootzone drying (PRD; Dry *et al.*, 1996; Kang and Zhang, 2004) that explicitly aim to vary soil moisture within part or all of the rootzone. Compared to crops grown with conventional deficit irrigation (DI, where water is applied to the entire rootzone), crops grown with PRD (the alternate irrigation and drying of only part of the root system) had significantly higher yield in six (out of 15) experiments (Dodd, 2009), but the physiological mechanisms underpinning these responses remain elusive.

PRD was conceived as an irrigation technique that aimed to alter root-to-shoot chemical signalling by drying part of the rootzone, thereby stimulating root synthesis of ABA and its subsequent transport to the shoot in order to partially close the stomata, thereby increasing leaf water use efficiency (Zhang and Davies, 1991; Stoll *et al.*, 2000; Kang and Zhang, 2004). Thus, the increased crop yields of PRD plants compared with DI plants may be partially attributed to the impacts of the drying and rewetting cycles (that characterize PRD) on root-to-shoot ABA signalling (Dodd *et al.*, 2006). However, relatively few studies have actually measured ABA concentrations in xylem sap or leaves of field-grown plants (but see Topcu *et al.*, 2007; Rodrigues *et al.*, 2008; Hutton and Loveys, 2011; Pérez-Pérez *et al.*, 2012; Romero *et al.*, 2012). Even under controlled environment conditions, PRD increased (Dodd, 2007), decreased (Dodd, 2007; Wang *et al.*, 2012) or had no consistent effect (Wang *et al.*, 2010, 2012) on xylem ABA concentration compared with DI plants, perhaps due to the timing of measurements during the drying/rewetting cycles. Taken together, these results suggest that the agronomic promise of PRD is unlikely to be consistently translated into improved crop water use efficiency in the field, unless irrigation managers can better predict its physiological effects.

Consequently, laboratory studies with ‘two root-one shoot’ grafted plants (Dodd, 2007) determined the contributions of different parts of the root system to total sap flow and leaf xylem ABA concentration ([X-ABA]_leaf_) during PRD, which better explained [X-ABA]_leaf_ than assuming it was determined by total soil water availability (Dodd *et al.*, 2008a, b, 2010). While these studies only exposed plants to a single soil drying cycle where the wet and dry parts of the rootzone remained the same (PRD-Fixed), in the field PRD usually alternates the wet and dry parts of the rootzone (Stoll *et al.*, 2000; Romero *et al.*, 2012). Soil drying and re-wetting cycles stimulated root growth (Mingo *et al.*, 2004), enhanced soil nutrient availability (Wang *et al.*, 2010), and altered root-sourced chemical signalling to the shoots by transiently increasing [X-ABA]_leaf_ (Dodd *et al.*, 2006; Romero *et al.*, 2012). However, it was not clear whether this increase was due to the remobilization of ABA that had accumulated in the previously dried rootzone, or due to the drying of previously irrigated roots.

Measuring and modelling xylem ABA concentrations (Dodd, 2008; Dodd *et al.*, 2008a, b, 2010; Liu *et al.*, 2008; Plauborg *et al.*, 2010) is challenging, due to the methodological difficulties of collecting an authentic xylem sap sample. In field-grown crops, root xylem sap can only be sampled by de-topping the plant and collecting sap at relatively low flow rates compared with whole plant transpiration rate, which artificially increases root xylem sap ABA concentration, [X-ABA]_root_ (Else *et al.*, 1994). Consequently, many studies have collected xylem sap from detached leaves or stems, by measuring their water potential and then applying an overpressure (Jachetta *et al.*, 1986; Dodd, 2007). While varying the overpressure applied to detached tomato leaves had minimal effects on [X-ABA]_leaf_ (Dodd *et al.*, 2009), actual concentrations can be higher than [X-ABA]_root_, depending on both the accuracy with which root xylem sap flow rate is matched with transpirational flow rate (Dodd *et al.*, 2008a; Netting *et al.*, 2012) and/or a dilution of leaf apoplastic sap with symplastic contents during sap collection (Jachetta *et al.*, 1986; Borel and Simonneau, 2002). For this reason, modelling xylem ABA concentrations of plants exposed to heterogeneous soil moisture may be more informative when a single xylem sap sampling methodology and/or site of xylem sap sampling (either root or leaf) is adopted.

This study aimed to predict the [X-ABA]_leaf_ of plants exposed to temporal and spatial differences in soil moisture imposed by fixed or alternate PRD. Whereas previous models of [X-ABA]_leaf_ (Dodd *et al.*, 2008a, b, 2010) relied on direct measurements of root xylem ABA concentrations from different parts of the root system, this study developed a model that collected xylem sap only from leaves (and thus could be applied to field-grown plants). Initial experiments with ‘two root-one shoot’ grafted sunflower plants determined whether sap flow gauges and soil moisture sensors gave similar relationships between the fraction of water uptake by roots in drying soil and soil water content (θ). Then water was withheld from the entire rootzone of own-rooted tomato plants to determine the relationship between [X-ABA]_leaf_ and θ, which was used to predict [X-ABA]_root_ from measurements of θ by assuming that [X-ABA] remained constant in transit from roots to shoots. Finally, different models were used to predict [X-ABA]_leaf_ of split-root tomato plants at different times in soil drying and re-wetting cycles during PRD. Whereas xylem ABA concentration during fixed PRD was related strictly to the soil water content of the irrigated rootzone, both drying and re-irrigated parts of the rootzone contributed following PRD alternation.

## Materials and methods

### Determining the contributions of different parts of the root system to total sap flow in ‘two root-one shoot’ plants exposed to a single PRD drying cycle

The initial experiments used ‘two root-one shoot’ sunflower (*Helianthus annuus* L. cv. Tall Single Yellow) plants since the grafting procedure gave higher success rates in this species and because the cylindrical stems of sunflower were ideal for measuring sap flow from different root systems. Seeds (Moles Seeds, Essex, UK) were placed on two layers of filter paper (Whatman No. 1) moistened with distilled water in a covered Petri dish and germinated in the dark for 48h. Five seedlings (typical radical length 20mm) were placed each side of a vertical, watertight plastic partition in a 3.0 l pot (17cm diameter, 13cm high) filled with an organic loam (John Innes No. 2, J Arthur Bowers, Lincoln, UK) substrate with a gravimetric water content (θ) at a drained capacity of 0.63g g^–1^. The substrate was watered to drained capacity prior to seedling placement, then pots were placed in a plastic container, the top of the container covered with aluminium foil (to exclude light and promote hypocotyl extension), and the container placed in a walk-in controlled environment room. After one week, the aluminium foil was removed and the plants grown for a further two weeks before ‘two root-one shoot’ grafting was implemented with uniform seedlings, as described previously (Dodd, 2007). A plastic bag was secured around the pot base with a rubber band, and the grafted plants, which resembled an inverted ‘Y’, were allowed to establish for 2 weeks. When the plastic bags were removed, only one grafted plant was allowed to grow in each pot. Plants were watered daily and allowed to grow for a further two weeks prior to experiments.

Plants were raised in a single walk-in controlled environment room (3×4 m) at the Lancaster Environment Centre under a 12h photoperiod (09.30–21.30h). Day–night variation caused fluctuations in temperature (16–26 °C) and atmospheric evaporative demand (0.2–1.2 kPa). Metal halide lamps (HQIT 400N, Osram, St Helens, UK) were 1.2 m above bench height and provided 220 µmol m^–2^ s^–1^ photosynthetic photon flux density (PPFD) at bench height.

**Fig. 1.**
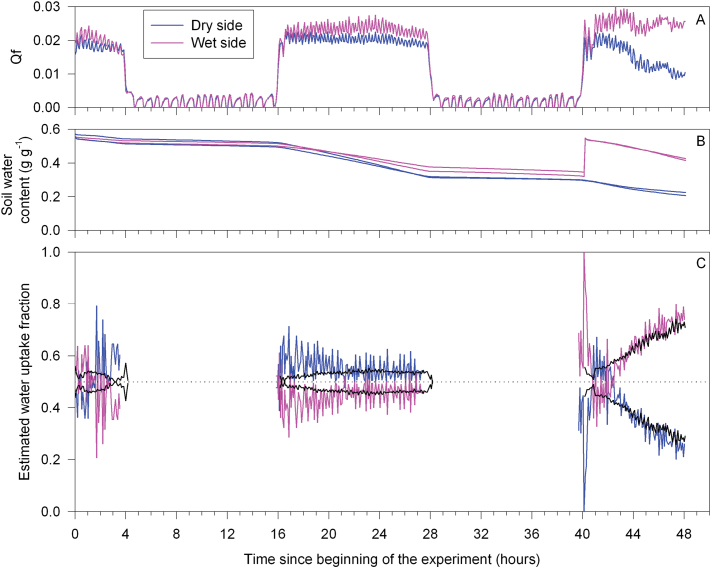
Heat loss by convection (*Q*_f_) measured by two sap flow gauges (A), soil water content measured by two theta probes per soil compartment (B), and the fractions of sap flow (black lines) and root water uptake (coloured lines) (C) from drying (blue) and irrigated (pink) parts of the rootzone (C) of a ‘two root-one shoot’ grafted sunflower plant. Non-continuous data in (C) indicate the night period.

Prior to sap flow measurements, the entire pot was watered to drained capacity. Sap flow through each hypocotyl (below the graft union) was measured using the heat balance technique with commercially available sensors (Model SGA-5, Dynagage^®^, Dynamax Inc, Houston, TX, USA) suitable for stems of 5–7mm diameter, which was directly measured above the sensor after installation. Sensor installation and operation were according to the manufacturer’s instructions (Dynagage, 2005). Foam (15mm thick) and aluminium foil shielded the sensors from direct radiation. Power input to the heater was constant for all measurements. Sap flow was recorded every 10 s and averaged over 5min using a datalogger (Model DL2e, Delta-T Devices, Cambridge, UK). For each hypocotyl, *Q*f (heat loss by convection by the sap; Fig. 1A) was expressed as a fraction of total *Q*f (Fig. 1C). Previous measurements established that total *Q*f (the sum of both hypocotyls) was temporally correlated with whole plant transpiration measured gravimetrically by placing the plant on a balance (Dodd *et al.*, 2008a).

After fitting the sap flow gauges, two theta probes (Model ML2X, Delta-T Devices) with 6.5cm pins were placed vertically into the top of each soil compartment to measure θ, and measurements recorded every 5min using a datalogger (Model DL2e, Delta-T Devices). During the experiments, water was supplied to one soil compartment (Figs 1B, 3B) to ensure that θ exceeded 0.3g g^–1^, corresponding to a soil matric potential of –0.1MPa (Dodd *et al.*, 2006).

### Modelling leaf xylem ABA concentration of own-rooted plants following PRD alternation

Subsequent experiments used tomato (*Solanum lycopersicum* Mill. cv. Ailsa Craig) as it was easier to collect sufficient xylem sap from individual detached tomato leaves. *S*eeds (Moles Seeds, Essex, UK) were individually sown in a well-watered peat-based substrate (Levingtons M3, Levington Horticulture Ltd., Ipswich, UK) in seedling trays, with a single seed in each separate compartment (30mm deep×20 mm×20mm). After 14 d, when the first true leaf had emerged, seedlings were transferred to ‘net pots’ (Teku, Pöppelmann Plastiques, Pöppelmann, France) of 50mm diameter×50mm deep with 5 mm×7mm pores in the sides. After one more week, plants in the ‘net pots’ were transplanted into custom-made 3.0 *L* split pots (17cm diameter, 13cm high). A vertical, watertight plastic partition separated two halves of the 3.0 l pot, with a gap (50mm deep×50mm wide) in the centre of the partition to allow each ‘net pot’ to be inserted into the substrate, minimizing seedling disturbance. Both ‘net pots’ and split pots were filled with an organic loam (John Innes No. 2, J Arthur Bowers, Lincoln, UK) and watered daily until each experiment commenced.

Several batches of tomato plants were produced as described above, with an initial experiment aiming to define relationships between [X-ABA]_leaf_ and whole pot θ and leaf water potential (Ψ_leaf_). Eight weeks after the seeds were planted, different irrigation treatments were applied by withholding irrigation from the entire pot (homogeneous irrigation, DI) or half of it (heterogeneous irrigation, PRD). To generate a range of whole-pot soil water contents, water was withheld for 24–48h, but all plants (15 for PRD and 7 for DI) were sampled on the same day (between 10 00h and 17 00h) by sequentially excising three fully expanded leaves (Leaves 5–7 numbering from the base of the plant) from each plant. Ψ_leaf_ was measured using a Scholander type pressure chamber (Plant Moisture Systems, Santa Barbara, CA, USA), then an overpressure (0.4MPa) was applied to the leaf to express xylem sap which was collected in a pre-weighed Eppendorf tube and frozen in liquid nitrogen for later determination of ABA concentration via radioimmunoassay (Quarrie *et al.*, 1988). Two measurements of soil water status of each compartment were made by inserting a theta probe (Model ML2X, Delta-T Devices) into the top of the pot, after which the soil (including roots and ‘net pot’) was carefully removed from the pot, weighed, and then oven-dried to determine θ.

To quantify the fractional soil water uptake from each compartment in subsequent experiments (each comprising four plants per week), two theta probes (Model ML2X, Delta-T Devices) were placed vertically into the top of each soil compartment to measure θ. Then water was supplied to only one soil compartment to implement partial rootzone drying (PRD-Fixed). After 3 d of soil drying and before the start of the photoperiod on the fourth day, the wet and dry soil compartments were alternated (PRD-Alternated) to compare the relationships between soil and plant variables with those plants where the wet and dry soil compartments were fixed. Fully expanded leaves (Leaves 5–7 numbering from the base of the plant) were detached to measure Ψ_leaf_ and to collect xylem sap as described above at the end of the third day of soil drying (PRD-Fixed, Leaf 5) and 2h (Leaf 6) and 6h (Leaf 7) after the wet and dry soil compartments were alternated. Preliminary experiments revealed that no more than three leaves could be harvested from the one plant without substantially affecting the relationship between [X-ABA]_leaf_ and soil water content (data not shown). An additional group of well-watered (θ >0.35g g^–1^) plants were sampled to compare the effects of the PRD-Fixed and PRD-Alternated treatments on whole pot soil water content (θ_pot_), Ψ_leaf_, and [X-ABA]_leaf_.

**Fig. 2.**
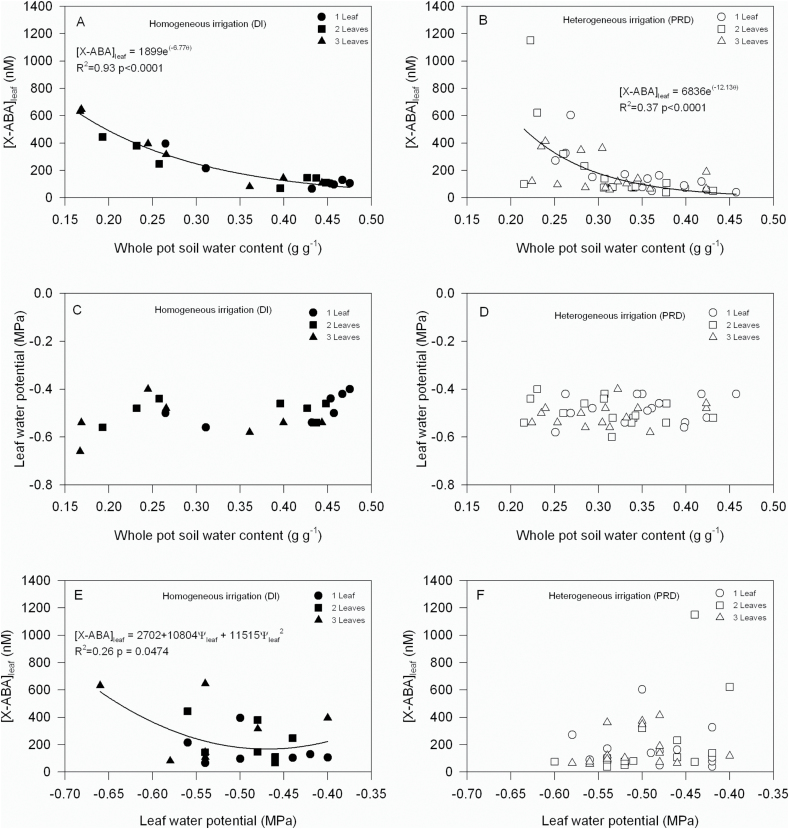
Relationships between leaf xylem ABA concentration ([X-ABA]_leaf_) and whole-pot soil-water content (A, B), leaf water potential and whole-pot soil water content (C, D), and [X-ABA]_leaf_ and leaf water potential (E, F) in tomato plants exposed to homogeneous (deficit irrigation; A, C, E) and heterogeneous (fixed PRD; B, D, F) irrigation. Each point represents an individual leaf (three leaves were sampled sequentially from each plant), with regression lines fitted where significant (*P* <0.05).

In an attempt to understand the variation in [X-ABA]_leaf_ generated in response to fixed and alternate PRD, measured [X-ABA]_leaf_ was compared with the [X-ABA]_leaf_ predicted from three models where:

(i) [X-ABA]_leaf_ depended only on whole pot soil water content (as in Fig. 2A) using the relationship:

$${\left[X-ABA\right]}_{\text{leaf}}=1899e^{-6.77\theta }$$ (1)

where θ is the mean soil water content, determined by theta probe measurements, derived by averaging both sides of the pot from DI plants.

(ii) [X-ABA]_leaf_ depended only on leaf water potential (as in Fig. 2E) using the relationship:

$${\left[X-ABA\right]}_{\text{leaf}}=-2702+10804\Psi_{\text{leaf}}+11515\Psi_{\text{leaf}}{}^{2}$$ (2)

where Ψ_leaf_ is the leaf water potential of an individual leaf from DI plants.

(iii) [X-ABA]_leaf_ depended on θ of each compartment of the split-pot, which affected both [X-ABA]_root_ emanating from, and soil water uptake by, roots in those compartments, according to a simple model (Dodd *et al.*, 2008a):

$${\left[X-ABA\right]}_{\text{leaf}}=F_{\text{wet}}{\left[X-ABA\right]}_{\text{root}-\text{wet}}+F_{\text{dr}y}{\left[X-ABA\right]}_{\text{root}-\text{dry}}$$ (3)

where *F*_dry_ and F_wet_ represent the fractions of sap flow, and [X-ABA]_root-wet_ and [X-ABA]_root-dry_ represent root xylem ABA concentrations from wet and dry parts of the root system, respectively. Since xylem sap was collected only from leaves in this study, [X-ABA]_root-wet_ and [X-ABA]_root-dry_ were simulated using equation 1, by assuming no change in [X-ABA] in transit between roots and shoots and considering only soil water content of wet or dry sides of the pot, respectively.

### Statistical analysis

Within an irrigation treatment (PRD-Fixed or PRD-Alternated) or combined across irrigation treatments, regression analysis determined the significance of relationships between soil and plant variables. Two-way analysis of variance (ANOVA) determined whether the wet or dry root system of PRD plants, or irrigation treatment, altered relationships between soil and plant variables. A change in the sensitivity of the *y*-variable to the *x*-variable is given by a significant interaction term (*x*-variable by treatment or part of the root system).

## Results

Before imposing a PRD treatment on a typical ‘two root-one shoot’ sunflower plant, sap flow through the two hypocotyls (Fig. 1A) and soil moisture of the two soil compartments (Fig. 1B) were similar. Even though soil moisture declined from ~0.51 to ~0.34g g^–1^ in both compartments during the second photoperiod, sap flow through both hypocotyls was maintained. At the beginning of the third photoperiod, one half of the pot was watered to impose PRD, thereby raising soil water content back to ~0.54g g^–1^ (Fig. 1B) which maintained sap flow through this part of the root system (Fig. 1A). Soil water content continued to decline in the dry side of the pot (albeit at a reduced rate) and sap flow from that root system started to decline, thus the fraction of total sap flow through the dry and wet root systems decreased and increased, respectively (Fig. 1C). A similar pattern was noted by calculating (from soil moisture readings) the fractions of water uptake from each side of the root system (Fig. 1C). Thus, proportional water uptake from different parts of the rootzone could be determined with sap flow gauges (only in grafted plants) or soil moisture sensors.

To determine the xylem ABA responses of own-rooted, ungrafted tomato plants at different times within soil drying and re-wetting cycles during PRD, it was necessary to collect xylem sap from different leaves of the same plant. Since the sensitivity of leaf xylem ABA concentration ([X-ABA]_leaf_) to soil water content (θ) and leaf water potential (Ψ_leaf_) was not significantly altered by the sequential removal of three leaves (Fig. 2), each plant could be sampled three times during PRD cycles.

Withholding irrigation from the entire rootzone, or half of it, produced an exponential relationship between [X-ABA]_leaf_ and whole-pot soil water content (θ_pot_) (Fig. 2A, B). Homogeneous or heterogeneous irrigation induced a similar response, as there was no difference in the slope of the relationship according to whether DI or PRD was applied (*P*=0.71). However, changes in θ_pot_ produced by DI or PRD were not correlated with Ψ_leaf_ (Fig. 2C, D). By contrast, the spatial distribution of irrigation altered the relationship between Ψ_leaf_ and [X-ABA]_leaf_ (a difference in the slope of relationship: *P*=0.028) since [X-ABA]_leaf_ correlated with Ψ_leaf_ only in homogeneously irrigated plants (Fig. 2E). The ability of these relationships between [X-ABA]_leaf_ and θ_pot_ (Fig. 2A; equation 1) and Ψ_leaf_ (Fig. 2E; equation 2) to predict [X-ABA]_leaf_ was tested in subsequent experiments that exposed own-rooted, ungrafted tomato plants to PRD for 3 d and sampled leaves on three occasions (before alternation as PRD-Fixed plants, and 2h and 6h after alternating the wet and dry parts of the rootzone as PRD-Alternated plants).

**Fig. 3.**
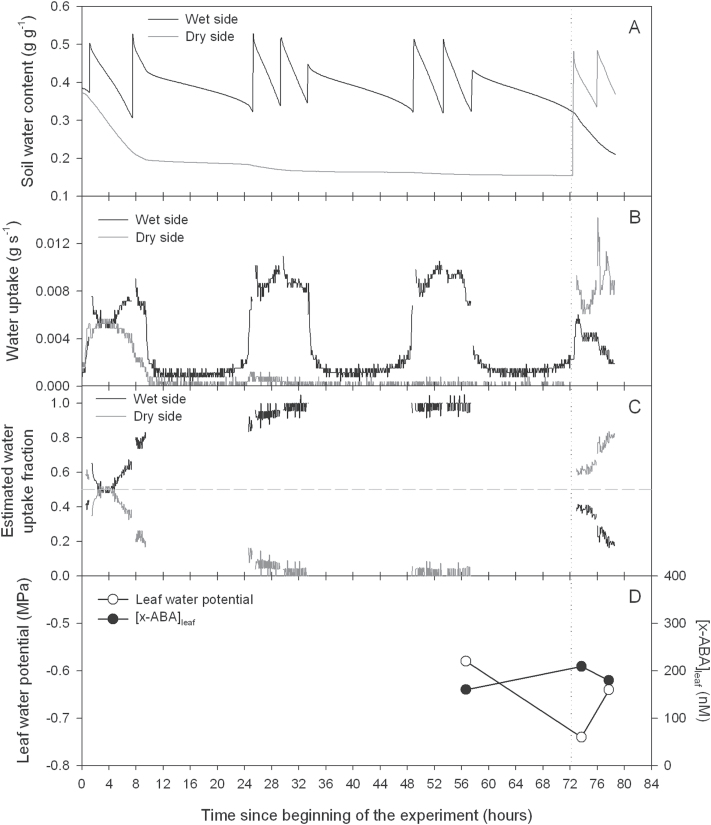
Soil water content (A) and water uptake (B) from two parts of the root system of tomato plants exposed to partial rootzone drying, which was used (C) to derive the fraction of water uptake from each part where the horizontal (dashed) line indicates an estimated water uptake fraction of 0.5. (D) Leaf water potential (open circles) and leaf xylem ABA concentration ([X-ABA]_leaf_: filled circles). Non-continuous data in (C) indicate the night period. After 3 d of PRD, the wet and dry sides of the root system were alternated (indicated by a vertical dotted line).

In a typical plant, frequent watering maintained θ of the wet (left) side of the pot greater than 0.3g g^–1^, but θ of the dry (right) side of the pot rapidly decreased below 0.2g g^–1^ once irrigation was withheld (Fig. 3A), such that water uptake from this compartment virtually ceased on the second day (Fig. 3B). At the end of the third day of drying, Ψ_leaf_ and [X-ABA]_leaf_ of this plant were –0.58MPa and 160nM, respectively (Fig. 3D), while θ of the wet and dry compartments were 0.35g g^–1^ and 0.16g g^–1^ (Fig. 3A) and the fractions of soil water uptake from these compartments were 0.98 and 0.02, respectively (Fig. 3C).

**Fig. 4.**
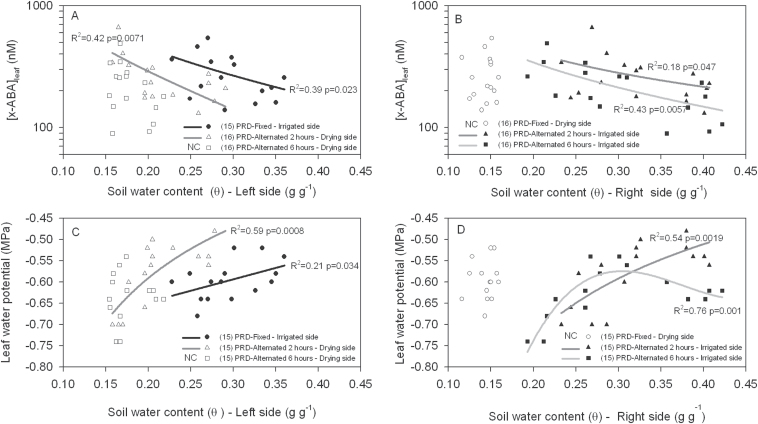
Relationships between leaf xylem ABA concentration ([X-ABA]_leaf_) (A, B) and leaf water potential (C, D) and soil water content from both sides (left and right) of the root system of tomato plants. In PRD-Fixed plants, left and right sides corresponded to wet and dry parts of the root system while, in PRD-Alternated plants, they corresponded to the newly-drying and just re-watered parts of the root system. In PRD-Alternated plants measurements were taken 2h and 6h after irrigation was alternated. Each point represents a single leaf and regression lines were fitted where significant (*P* <0.05). NC means not correlated.

Similar measurements were made at this time in the PRD cycle in 11 other plants, when the average θ of the wet and dry sides were 0.30g g^–1^ and 0.14g g^–1^, respectively, while the fractions of soil water uptake from these compartments in these PRD-Fixed plants were 0.98 and 0.02, respectively (Table 1). Across all plants in this study, average (mean of 12 plants) Ψ_leaf_ and [X-ABA]_leaf_ of PRD-Fixed plants were –0.60MPa and 273nM, respectively (Table 2). At this time, Ψ_leaf_ was negatively and [X-ABA]_leaf_ positively correlated with θ of the irrigated compartment (Fig. 4A, C). Predicting [X-ABA]_leaf_ based on either whole pot θ (equation 1) or Ψ_leaf_ (Equation 2) overestimated its value by 75% and 54%, respectively (Table 3). Multiplying the fraction of soil water uptake from each compartment by the predicted [X-ABA]_root_ based on its θ, and summing these terms (Equation 3), underestimated [X-ABA]_leaf_ by only 10% (Table 3).

**Table 1.** Average of soil water content and estimated water uptake fraction for each side of the pot (right and left) of fixed and alternated PRD tomato plants

Soil water content θ (g g^–1^)			
Irrigation treatment	Right side	Left side	*P*-value
PRD-Fixed	0.30±0.05	0.14±0.01	< 0.0001
PRD-Alternated 2 h	0.22±0.04	0.32±0.06	< 0.0001
PRD-Alternated 6 h	0.18±0.02	0.31±0.08	< 0.0001
**Estimated water-uptake fraction**			
**Irrigation treatment**	Right side	Left side	*P*-value
PRD-Fixed	0.98±0.02	0.02±0.02	< 0.0001
PRD-Alternated 2 h	0.28±0.18	0.72±0.18	< 0.0001
PRD-Alternated 6 h	0.10±0.08	0.90±0.08	*< 0.0001*

Data are means ±SE (*n*=12).

**Table 2.** Whole pot soil water content (θ_pot_), leaf xylem ABA concentration ([X-ABA]_leaf_), and leaf water potential (Ψ_leaf_) of well-watered, fixed, and alternated PRD tomato plants Within each column, different letters indicate significant differences between means at *P* ≤0.05 by Duncan’s test, with *P* values for 1-way ANOVA indicated.

Irrigation treatment	θ_pot_	[X-ABA]_leaf_	Ψ_leaf_
	(g g^–1^)	(nM)	(MPa)
Well watered	0.45±0.02 a	101±36 b	–0.50±0.06 a
PRD-Fixed	0.22±0.03 c	273±118 a	–0.60±0.04 b
PRD-Alternated 2 h	0.27±0.05 b	270±130 a	–0.59±0.08 b
PRD-Alternated 6 h	0.24±0.05 c	225±108 a	–0.63±0.06 b
*P*-value	<0.0001	< 0.0001	<0.0001

Data are means ±SE (*n*=12).

**Table 3.** The ability of different models to predict leaf xylem ABA concentration ([X-ABA]_leaf_) of fixed and alternated PRD tomato plants [X-ABA]_leaf_ was measured in detached leaves. For each plant, the difference between model (equations 1–3) and measurement is calculated as the ratio [X-ABA]_model_/[X-ABA]_leaf_. Values above or below 1 indicates that the model overestimates or underestimates [X-ABA]_leaf_, respectively. Three different models (equations 1–3) are indicated: see the Materials and methods. The numbers in the brackets are the *n* values.

Irrigation treatment	Mean (equation 1)	Leaf water potential (equation 2)	Fractional (equation 3)	*P*-value^*†*^
PRD-Fixed	$1.75_{a}^{A}$ (12)	$1.54_{b}^{AB}$ (12)	1.10^B^ (12)	0.030
PRD-Alternated 2 h	1.12_b_ (12)	1.50_b_ (12)	1.03 (12)	0.253
PRD-Alternated 6 h	$1.65_{a}^{A}$ (12)	$2.44_{a}^{A}$ (12)	1.31^B^ (12)	0.007
*P*-value^*‡*^	**0.012**	**0.044**	**0.186**	
Combined data	1.51^A^ (36)	1.83^A^ (36)	1.15^B^ (36)	0.001

^*†*^ Within each row (superscript capital letters) different letters indicate significant differences between means at *P* ≤0.05 by Duncan’s test.

^*‡*^ Within each column (subscript lowercase letters), different letters indicate significant differences between means at *P* ≤0.05 by Duncan’s test.

Returning to the typical plant, the dry (right) side of the pot was re-watered at the beginning of the fourth day and irrigation withheld from the previously irrigated (left) side (Fig. 3A). After alternating irrigation, soil water uptake increased progressively in the re-watered (right) side, while it decreased in the newly-drying (left) side (Fig. 3B). Two hours after alternation, Ψ_leaf_ and [X-ABA]_leaf_ of this plant were –0.74MPa and 209nM, respectively (Fig. 3D), while θ of the wet and dry sides were 0.40g g^–1^ and 0.29g g^–1^ (Fig. 3A) and the fractions of soil water uptake from these sides were 0.61 and 0.39, respectively (Fig. 3C).

Similar measurements were made at this time in the PRD cycle in 11 other plants, when the average θ of the newly irrigated (right) and now drying (left) sides were 0.32g g^–1^ and 0.22g g^–1^, respectively, while the fractions of soil water uptake from these compartments in these PRD-Alternated plants were 0.72 and 0.28, respectively (Table 1). Across all plants in this study, average Ψ_leaf_ and [X-ABA]_leaf_ of PRD-Alternated plants were –0.59MPa and 270nM, respectively (Table 2). At this time, variations in Ψ_leaf_ and [X-ABA]_leaf_ were correlated with θ of both sides of the pot (Fig. 4). Predicting [X-ABA]_leaf_ based on either whole pot θ (equation 1) or Ψ_leaf_ (equation 2) overestimated its value by 12% and 50%, respectively (Table 3). Multiplying the fraction of soil water uptake from each compartment by the predicted [X-ABA]_root_ based on its θ, and summing these terms (equation 3), underestimated [X-ABA]_leaf_ by only 3% (Table 3).

Returning again to the typical plant 6h after alternation, soil water uptake from the drying (left) side of the pot continued to decrease (Fig. 3B). At this time, Ψ_leaf_ and [X-ABA]_leaf_ of this plant were –0.64MPa and 180nM, respectively (Fig. 3D), while θ of the wet and dry sides were 0.40g g^–1^ and 0.22g g^–1^ (Fig. 3A) and the fractions of soil water uptake from these sides were 0.80 and 0.20, respectively (Fig. 3C).

Similar measurements were made at this time in the PRD cycle in 11 other plants, when the average θ of the newly irrigated (right) and now drying (left) sides were 0.31g g^–1^ and 0.18g g^–1^, respectively, while the fractions of soil water uptake from these compartments in these PRD-Alternated plants were 0.90 and 0.10, respectively (Table 1). Across all plants in this study, average Ψ_leaf_ and [X-ABA]_leaf_ were –0.63MPa and 225nM, respectively (Table 2). At this time, variations of [X-ABA]_leaf_ and Ψ_leaf_ were only correlated with changes of θ from the newly-irrigated (right) side (Fig. 4B, D). Predicting [X-ABA]_leaf_ based on either whole pot θ (equation 1) or Ψ_leaf_ (equation 2) overestimated its value by 1.6-fold (~60%) and 2.4-fold, respectively (Table 3). Multiplying the fraction of soil water uptake from each compartment by the predicted [X-ABA]_root_ based on its θ, and summing these terms (equation 3), overestimated [X-ABA]_leaf_ by only 31% (Table 3). Therefore at all stages of the PRD cycle, equation 3 best estimated [X-ABA]_leaf._

**Fig. 5.**
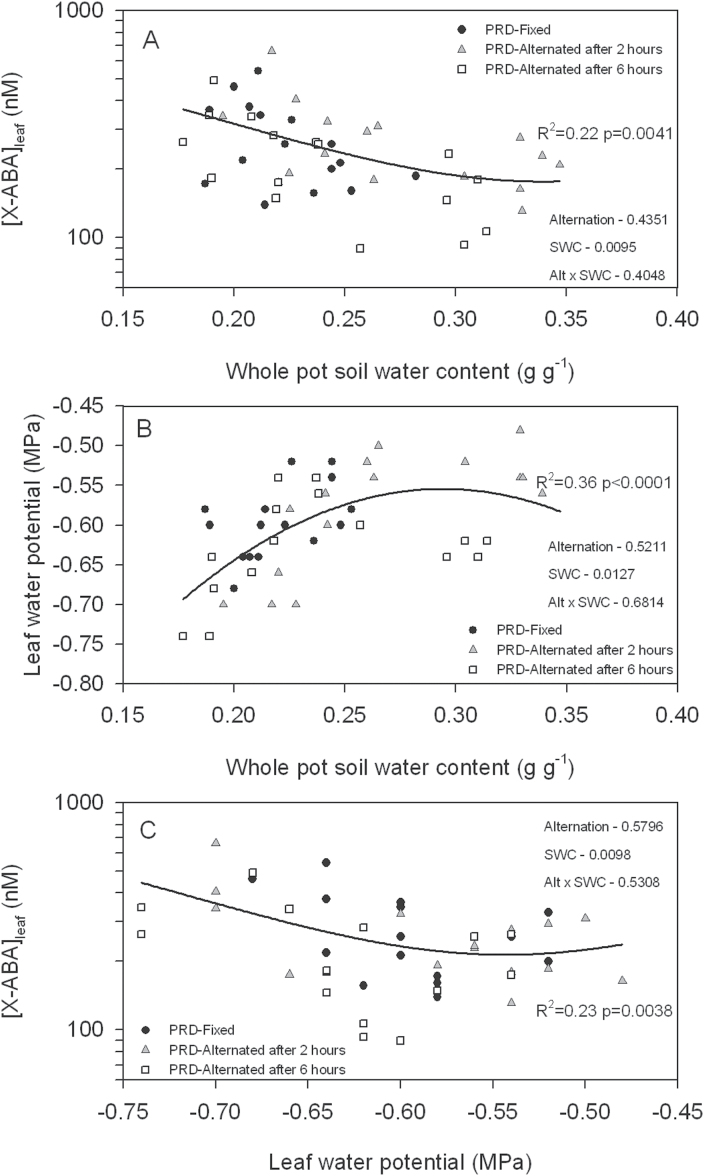
Relationships between (A) [X-ABA]_leaf_ and whole-pot soil water content, (B) leaf water potential and whole-pot soil water content and, (C) leaf xylem ABA concentration ([X-ABA]_leaf_) and leaf water potential for PRD-Fixed and PRD-Alternated (after 2h and 6h) tomato plants. *P*-values determined by two-way analysis of variance (ANOVA) for irrigation treatment (PRD-Fixed and PRD-Alternated—combining data from 2h and 6h after alternation), *x*-variables and their interaction are presented. Each point represents a single leaf measurement and regression lines were fitted where significant (*P*<0.05).

Applying PRD-Fixed and PRD-Alternated irrigation decreased θ_pot_ compared with well-watered plants, with θ_pot_ lower when heterogeneous soil moisture conditions were clearly established (Table 2). In both PRD-Fixed and PRD-Alternated plants, Ψ_leaf_ decreased and [X-ABA]_leaf_ increased similarly compared with well-watered plants (Table 2). It was also of interest to determine whether PRD alternation affected the relationships between [X-ABA]_leaf_, θ_pot_, and Ψ_leaf_. When θ_pot_ decreased below 0.26g g^–1^, Ψ_leaf_ declined similarly in PRD-Fixed and PRD-Alternated plants (Fig. 5B). When θ_pot_ exceeded 0.26g g^–1^, PRD-Alternated plants had a lower Ψ_leaf_ 6h after alternation of the wet and dry sides than after 2h. [X-ABA]_leaf_ increased as Ψ_leaf_ declined (Fig. 5C) or as θ_pot_ (Fig. 5A) increased, but the timing (2h and 6h after alternation) or occurrence (PRD-Fixed versus PRD-Alternated) of PRD alternation did not affect the sensitivity of ABA signalling. Generally, all PRD plants had similar relationships between [X-ABA]_leaf_, θ_pot_, and Ψ_leaf_ independently of alternating the wet and dry sides of the pot.

## Discussion

The irrigation technique of partial rootzone drying was conceived as a field adaptation of laboratory split-root soil drying experiments, to enhance root-to-shoot chemical signalling to improve crop water use efficiency by causing partial stomatal closure and decreasing excessive vegetative vigour (Dry *et al.*, 1996; Kang and Zhang, 2004). While PRD can outyield conventional deficit irrigation (DI) where the entire rootzone is irrigated (Dodd, 2009), understanding the physiological mechanisms underpinning this technique has received comparatively little attention. Although many authors have postulated a role for root-sourced ABA in causing these physiological responses, there is considerable variability in the relative response of [X-ABA]_leaf_ when comparing PRD and DI plants (Dodd, 2007; Wang *et al.*, 2012), perhaps related to the timing of measurements during drying/re-wetting cycles (Dodd *et al.*, 2006) and/or total soil water availability (Romero *et al.*, 2012). Although models have demonstrated the importance of sap flow from roots in drying soil in predicting [X-ABA]_leaf_ of PRD plants (Dodd *et al.*, 2008a, b, 2010), these require [X-ABA]_root_ as an input variable, making them difficult to apply to field situations. By collecting xylem sap only from detached leaves and measuring water uptake by different parts of the root system, this study developed a model, suitable for field application, that better predicted [X-ABA]_leaf_ of PRD plants (throughout drying and rewetting cycles) than assuming that [X-ABA]_leaf_ was simply related either to total soil water availability or to leaf water potential.

In previous studies, [X-ABA]_leaf_ was predicted by quantifying both the fractions of sap flow, and root xylem ABA concentrations, from different parts of the root system using specially constructed ‘two root-one shoot’ grafted plants (Dodd *et al.*, 2008a, b, 2010), and assuming [X-ABA] was not altered in transit from roots to shoot. However, a major limitation of applying this model to field-grown, own-rooted plants exposed to PRD is the difficulty of determining the fraction of sap flow from each side of the root system. Continuous soil moisture monitoring can infer plant water uptake (Puertolas *et al.*, 2013), which was correlated with sap flow from different parts of the root system in ‘two root-one shoot’ grafted plants (Fig. 1). After withholding water from part of the rootzone, root water uptake and sap flow declined similarly as the soil dried. Thus soil-moisture sensors can determine when sap flow from the drying side of the root system ceases, although vertical gradients in soil moisture (and root water uptake) may complicate interpretation (Puertolas *et al.*, 2013).

Another complexity of modelling [X-ABA]_leaf_ during PRD is the periodic alternation of wet and dry sides, requiring repeated measurements from single plants to test the adequacy of models at different stages of the drying cycles. To monitor the effects of soil drying/re-wetting cycles on the [X-ABA]_leaf_ of individual plants, three leaves were sampled (Fig. 3D). Preliminary experiments established that relationships between [X-ABA]_leaf_ and both plant (leaf water potential) and soil water status (Fig. 2) did not vary with the number of leaves (per plant) sampled when plants were allowed to dry the soil in a split pot uniformly. Nevertheless, heterogeneous soil moisture increased the variation of [X-ABA]_leaf_ (from 60–1150nM in PRD plants and from 215– 645nM in DI plants across a similar θ and Ψ_leaf_ range; Fig. 2) as observed previously in tomato (Dodd, 2007), probably due to spatial differences in root water uptake when θ of the dry side of the pot decreased below 0.3g g^–1^ (Fig. 3). An additional contributing factor may be xylem sectoriality in this species (Zanne *et al.*, 2006), with specific roots supplying water (and possibly chemical signals) to specific leaves in the shoot, but the leaf sampled did not affect the relationship between [X-ABA]_leaf_ and θ in PRD plants (Fig. 2B), probably because roots were stochastically distributed between the two soil compartments of the split pot. Since roots of PRD plants are exposed to a greater range of soil moisture at a given θ_pot_ than DI plants, different water uptake fractions from each part of the root system can affect [X-ABA]_leaf_.

Based on previous studies with PRD plants, the optimal moment to alternate irrigation is when sap flow from the dry rootzone significantly decreases; thus limiting ABA export from roots to shoots (Dodd *et al.*, 2008*a*). When soil moisture was clearly heterogeneous (6h after alternation and during fixed PRD), water uptake from the dry rootzone practically ceased (Fig. 3C; Table 1). Under these conditions, variations of Ψ_leaf_ and [X-ABA]_leaf_ were mostly related to changes in the θ of the irrigated rootzone (Fig. 4), as previously modelled (Dodd *et al.*, 2008a, b). Furthermore, soil water status of this compartment is important to maintain high plant water status during PRD (Wang *et al.*, 2012). At these times of the PRD cycle, [X-ABA]_leaf_ was best predicted with a model that included the water-uptake fractions from each part of the root system (Table 3). As previously observed (Dodd *et al.*, 2008b), predicting [X-ABA]_leaf_ based on the predetermined relationship between [X-ABA]_leaf_ and whole pot θ (average of dry and wet sides of the pot, equation 1) significantly overestimated [X-ABA]_leaf_ (Table 3). Similarly, predicting [X-ABA]_leaf_ based on Ψ_leaf_ (equation 2) also substantially overestimated its concentration (Table 3). Thus accounting for soil water uptake from different parts of the root system best predicted [X-ABA]_leaf_ once water uptake from the dry rootzone had declined to 10% or less (Table 1), but these conditions may occur for limited periods of time in field-grown plants due to the availability of soil moisture at depth.

Two hours after re-watering the previously dry rootzone, water uptake from both soil compartments significantly contributed to the total sap flow (Fig. 3; Table 1) and thus [X-ABA]_leaf_. In this case, whole pot θ alone (equation 1) and accounting for water uptake from different parts of the rootzone (equation 3) showed a statistically similar ability to predict [X-ABA]_leaf_ (Table 3). By contrast, in an experiment where the fraction of soil water uptake from the drying compartment remained greater than 25% for four days of the five days of a PRD cycle, relating [X-ABA]_leaf_ to total soil water availability (and ignoring relative water uptake from the two compartments) was the better performing model (Liu *et al.*, 2008). However, the reliability of this conclusion depended on the range of soil water availability considered (Dodd *et al.*, 2008b), demonstrating that preliminary experiments to parameterize any model must occur over a similar range of soil water contents as tested experimentally (cf. Fig. 2A, C and Fig. 5A, B). An alternative view, that [X-ABA]_leaf_ can be predicted from Ψ_leaf,_ has received comparatively little attention since the relationship between [X-ABA]_leaf_ and Ψ_leaf_ varied according to whether plants received PRD or DI (Dodd, 2007; Dodd *et al.*, 2008a; cf. Fig. 2E and 2F here) and whether PRD was alternated or fixed (Dodd *et al.*, 2006) and sometimes there was no significant relationship between these variables. Accordingly, predicting [X-ABA]_leaf_ from Ψ_leaf_ (equation 2) systematically overestimated [X-ABA]_leaf_ at all stages of the PRD cycle (Table 3), suggesting again that accounting for soil water uptake from different parts of the root system best predicted [X-ABA]_leaf_, when soil moisture heterogeneity existed.

After alternating the wet and dry soil compartments of the pot, [X-ABA]_leaf_ was similar to fixed PRD plants (Table 2), contrary to previous observations. Greater [X-ABA]_leaf_ of alternated plants compared with fixed plants (Dodd *et al.*, 2006—tomato grown in the same substrate as in this study) was suggested to result from mobilizing root-sourced ABA (that had accumulated during soil drying) to the transpiration stream following re-watering the originally dry column. Two hours after irrigation alternation, variations of [X-ABA]_leaf_ were related to changes in soil water status of both sides of the pot (Fig. 4A, B). At this moment, water uptake from the previously dry side of the pot was re-established (Table 1), yet [X-ABA]_leaf_ did not show any unexpected increase due to ‘extra ABA’ transported from the previously dried roots. Reconciling these apparently contradictory observations requires more detailed information on the sensitivity of root ABA accumulation in response to soil drying. However, when there were more pronounced vertical gradients in soil moisture [as probably occurred in Dodd *et al.* (2006) where plants were grown in 30cm high soil columns], there was less pronounced root ABA accumulation when soil moisture was 0.13–0.25g g^–1^ compared with a more homogeneous soil-moisture distribution (Puertolas *et al.*, 2013) which characterizes the soil environment in the 13cm high pots used here. Instead, differences in the pot surface area-to-volume ratio in the soil compartments in the two studies (cf. Dodd *et al.*, 2006 versus this study) will expose different numbers of roots to different environmental (and soil-moisture) conditions at the edge of the pot. Clearly, more attention must be given to measuring root ABA accumulation in the field in attempting to explain why PRD alternation only sometimes stimulates xylem ABA concentration (cf. Pérez-Pérez *et al.*, 2012 versus Romero *et al.*, 2012).

In summary, soil moisture sensors accurately estimated water-uptake fractions from different parts of the rootzone in plants grown in split pots. This information improved the prediction of [X-ABA]_leaf_ in plants exposed to fixed and alternate PRD, compared with prediction based on total soil-water availability or leaf water potential alone. Further work is required to establish why PRD alternation did not enhance [X-ABA]_leaf_ (Fig. 5), contrary to previous work, and whether any changes in root-to-shoot ABA signalling are related to crop yields.
